# Public and industry knowledge and perceptions of US swine industry castration practices

**DOI:** 10.1017/awf.2023.99

**Published:** 2023-12-22

**Authors:** JM Neary, AP Guthrie, L Jacobs

**Affiliations:** 1 School of Animal Sciences, Virginia Tech, 175 West Campus Drive, Blacksburg, Virginia 24061, USA; 2 Department of Statistics, Virginia Tech, Blacksburg, VA, USA, 250 Drillfield Drive, 24061

**Keywords:** animal welfare, consumer and citizen perception, piglet, questionnaire, surgical castration, swine industry

## Abstract

In the United States (US), surgical castration of male piglets is typically performed without any form of analgesia. This may raise concerns with the public; however, there is no information regarding current public knowledge on swine industry practices in the US. In this study we gained insight into public knowledge and perception on castration with and without analgesia in comparison to knowledge of industry stakeholders on these same topics. Through an online survey, 119 respondents were asked four questions about castration in the US swine industry. Industry respondents were contacted via social media and networking. The general public sample was accessed through Mechanical Turk. Survey responses were categorised by experience (industry vs public). Industry respondents were more aware of practices compared to the general public. Most public respondents were unaware of castration practices and the lack of analgesia use. Respondents from rural communities were more aware of castration practices than (sub)urban communities and more aware of analgesia use than those from urban communities. Those with more education had greater awareness of castration practices (occurrence not frequency). Based on the results from this first US sample, knowledge on industry practices was especially lacking for public respondents, but also for a minority of industry respondents, indicating opportunities for education and further research on the topic.

## Introduction

Male piglets (*Sus scrofa domesticus*) intended for slaughter are surgically castrated in the United States (US) swine industry (Rault *et al.* 2011). Prior to 14 days of age, the procedure is performed without analgesia or anaesthesia and involves manual restraint of the boar, two incisions on the scrotum, removal of the testicles, and a cut or tear of the spermatic cord (American Veterinary Medical Association [AVMA] Welfare Division 2013). Castrations are performed by a trained technician and can take approximately 30 s. Castration is performed to prevent both aggressive behaviours later in life (Rydhmer *et al.* 2006), and boar taint in pork (AVMA Welfare Division 2013). Boar taint is caused by the post-pubertal deposition of androsterone and skatole in body fat, causing the meat to have an abnormal and somewhat foul odour when cooked (Keenan 2016). Prevalence of boar taint in pork ranges from 10–50% depending on factors such as age, breed, and environment (Prusa *et al.* 2011; Aluwé *et al.* 2015; Channon *et al.* 2018). Consumer sensitivity to boar taint ranges from 11–75% depending on factors such as the consumer’s age, gender, and geographic location (Bañón *et al.* 2004; Blanch *et al.* 2012; Channon *et al.* 2018). According to the US Food Safety and Inspection Service, carcases with boar taint should be condemned (9 CFR 311.20; Sexual Odor of Swine; United States Federal Government 2012). Producers surgically castrate boars to ensure boar taint does not develop and to avoid subsequent condemnation.

Public awareness of castration practices could incentivise producers to pay for additional costs related to analgesia use or apply alternative methods to avoid boar taint. One feasible alternative to castration is immunocastration. The boar receives two injections of a protein compound that induces the production of antibodies against gonadotropin-releasing hormone, which temporarily suppresses testicular development and function and, in turn, the production of androsterone and skatole (for a review, see AVMA 2013). While there is an immunocastration product that is approved by the US Food and Drug Administration (FDA) (AVMA Welfare Division 2013), there are no data available regarding usage in the US. Immunocastration is reportedly used on 3% of all males in the European Union (De Briyne *et al.* 2016) and has been the sole form of castration in New Zealand and Australia since 1998 (AVMA Welfare Division 2013). Surgical castration with anaesthesia is already used in The Netherlands, Germany, Norway and Switzerland (Vanhonacker *et al.* 2009) while another alternative would be to raise entire males and slaughter at a prepubertal weight (prior to seven months of age) as is the case in the Republic of Ireland, Portugal, Spain, The Netherlands and the United Kingdom (De Briyne *et al.* 2016). However, with the US being the third largest consumer, and one of the largest exporters of pork in the world (United States Department of Agriculture Economic Research Service [USDA ERS] 2019), this could present financial and logistical challenges within the supply chain. Another alternative to current surgical castration practices would be to provide analgesia during castration. However, there are currently no analgesic pharmaceuticals approved by the FDA for use in pigs (Bates *et al.* 2014).

No studies have considered US public perceptions of current castration practices. The public is generally unaware of animal husbandry (Alonso *et al.* 2020) and castration practices. Only 40% of consumers in Flanders, Belgium were aware of current castration practices in Belgium (Vanhonacker *et al.* 2009) while Brazilian citizens were unaware of castration of male pigs and the relationship between boar taint in pork and castration practices (Yunes *et al.* 2019; Hötzel *et al.* 2020). Similar results were found for European consumers (Vanhonacker & Verbeke 2011; Heid & Hamm 2013). A pooled survey of consumers from Belgium, France, Germany, and The Netherlands indicated that only 46% of respondents were aware of the existence of boar taint, and 51% aware of surgical castration (Vanhonacker & Verbeke 2011). German consumers of certified organic products were generally unaware of the connection between intact boars and meat quality but expressed interested in at least trying a ‘tainted’ product before deciding on future purchasing (Heid & Hamm 2013). With alternatives to surgical castration becoming increasingly feasible for industry application, it is worthwhile determining the knowledge and perceptions of the public regarding current swine industry castration practices. In Europe, surgical castration without analgesia was found to be the least accepted method (32%) (Aluwé *et al.* 2020). In comparison, there was high acceptance of castration with anaesthesia (85%), immunocastration (71%), and raising intact males (49%) (Aluwé *et al.* 2020). Since consumer pressures could result in changes in legislation or industry standards, this study aimed to use and online survey to perform an initial characterisation of both general public and industry stakeholder knowledge regarding current US swine castration practices.

## Materials and methods

All procedures were approved by Virginia Tech Human Research Protection Program Institutional Review Board, protocol #20-404.

### Sample

An online Qualtrics survey (SAP, Provo, UT, USA) was distributed in August and September 2020 with the aim of receiving responses from experienced swine industry respondents (‘industry’) and respondents from the general public (‘public’) with an equal number of responses from both groups (roughly a 1:1 ratio). Industry respondents were recruited via Facebook and by direct email to industry stakeholders and university faculty within the authors’ network across the US. Facebook posts were non-sponsored and not distributed in any Facebook groups. Facebook and swine industry contacts as well as university staff were invited to share the survey with others, including farm owners, operators, technicians and veterinarians. This method of non-probability snowball-sampling involves initial contact persons being asked to complete the survey together with the request to forward it to acquaintances, similar to Vanhonacker *et al.* (2009). A limitation with this is that non-probability sampling methods often result in biased samples that limit generalisability (Blair & Blair 2015). Public respondents were recruited using Amazon Mechanical Turk (Amazon Web Services, Seattle WA, USA). Amazon Mechanical Turk is a crowdsourcing platform where people receive pay to perform virtual tasks. This method of recruitment has been deployed successfully in the past (e.g. Sato *et al.* 2017). Public respondents received a monetary compensation through the website, depending on the mean duration required to complete the survey. Industry respondents did not receive compensation. All respondents were aged 18 years or older and lived in the US and responses were entered anonymously. Survey respondents were requested to provide informed consent prior to accessing the survey tool and the consent form is provided as supplementary material in Neary *et al.* (2022).

One hundred and twenty-nine completed surveys were received. Five of these were omitted due to the respondents not living in the US, and a further five were left out for failing an attention check question. Survey respondents were categorised based on their experience in the swine industry. Public respondents had no professional swine industry experience, and industry respondents had professional, paid swine industry experience. We included 119 completed surveys in the analysis, 66 from public respondents (55%) and 53 from industry respondents (45%).

### Survey instrument

The data presented here originate from a larger survey (up to 76 questions depending on the respondents’ answers, i.e. respondents without industry experience were not asked for details about their industry experience). One section on the applicability of the Piglet Grimace Scale is presented elsewhere (Neary *et al.* 2022). Three of the survey’s five sections consisted of: (1) demographic questions (up to 12 questions depending on their responses); (2) questions on agricultural and swine industry experience (up to 12 questions depending on their responses); and (3) four questions about perception and knowledge on castration and analgesia procedures. The entire survey took a mean (± SD) of 18 (± 24) min to complete, however this value excludes two survey entries that were completed over 24 h after starting the survey.

### Demographic questions

The first section of the survey included questions about gender (male, female, non-binary, prefer to self-describe, prefer not to say), age category (18–25, 26–35, 36–45, 46–55, 56–65, 66+ years old), home state or territory, community type (city/urban, suburban, rural, other), and highest level of education. Based on home state or territory entries, respondents were categorised into one of five regions in the US, including Northeast, Southeast, Midwest, West, and Southwest.

### Agriculture and swine industry experience

The second section of the survey included questions regarding current and previous experience with agriculture and swine. These included questions about frequency of visits to an animal production farm in the last twelve months (daily, weekly, monthly, less than monthly but more than once, once, never) and about their work experience with agriculture. If respondents indicated they had agriculture experience, questions followed on their experience with animal agriculture and animal species. If respondents had experience with swine, they were asked about the duration of professional work experience (no professional experience, less than 1 year, 1–5 years, 6–10 years, 11+ years).

### Perception and knowledge of swine industry castration practices

The main section of the survey consisted of four questions regarding the respondents’ perceptions and knowledge of common castration procedures within the US swine industry. The first question (Q1) pertained to whether respondents had been aware that castration may occur in the US swine industry prior to the survey, and how many males would undergo the procedure (Q2), which was expressed as % of males (never, 1–25% of males, 26–50% of males, 51–75% of males, 76–99% of males, or all males). The third question (Q3) asked if any type of pain relief (anaesthesia or topical analgesia) was used. Finally, respondents were asked (Q4) how many males would receive analgesia (never, 1–25% of castrated males, 26–50% of castrated males, 51–75% of castrated males, 76–99% of castrated males, all castrated males).

### Statistical analysis

The impact of Industry Experience, Community Type, Education Level, and Professional Swine Experience on knowledge and perception of swine castration was assessed using regression modelling. Each predictor was assessed separately using logistic regression models for Q1 and Q3 and using ordinal logistic regression models for Q2 and Q4.

To improve the stability of the models, categories for Education Level and Professional Swine Experience were collapsed for these analyses. Education Level was collapsed into three categories: No College Degree (High school degree/GED, Some college [no degree]), Undergraduate Degree (two-year or four-year degree), and Graduate/Professional Degree (Masters’ degree, Professional degree, or Doctorate [PhD]). Professional Swine Experience was collapsed into four categories: No Experience, < 1 year, 1–5 years, and 6+ years (6–10 years or 11+ years).

One limitation of this analysis is that the limited sample size prohibited us from fitting one model with all four predictors. The limited sample size caused quasi-separation issues in which the model perfectly predicted some categories but could not produce stable estimates (Hosmer & Lemeshow 2000). To mitigate this, we fitted separate models for each of the four predictors and collapsed the categories of predictors as described above. The logistic regression models for Q1, Q2, and Q3 with Professional Swine Experience as a predictor remained unstable because all respondents selected the same survey answer. Therefore, odds ratios are not reported in these cases. Rather, a Fisher’s Exact Test was run to assess the relationship between Professional Swine Experience and the survey question of interest.

All logistic regression models and corresponding odds ratios, predicted probabilities, and confidence intervals were generated using SAS software, Version 9.04.01 via SAS Studio, Release 3.81 (SAS Institute Inc, Cary, NC, USA 2020). The Fisher’s Exact Tests were run using JMP Pro software, version 16.1.0 (SAS Institute Inc, Cary, NC, USA 2021).

Odds ratios with corresponding 99% confidence intervals and *P*-values are reported. Additionally, predicted probabilities of responses from each predictor category with corresponding 99% confidence intervals are reported. The use of a 0.01 significance level rather than 0.05 is to reduce the risk of a false positive, given that we were running a large number of statistical tests.

## Results

### Respondent characteristics

Survey respondents’ demographics are presented in Table 1. Forty-five percent of public respondents had visited an animal production facility at some point in the last year and 15% of public respondents indicated non-professional experience with swine. Fourteen percent of public respondents did not respond to the question regarding their experience with castration. The majority of industry respondents (85%) visited an animal production facility monthly or more frequently in the last year, while three respondents had not visited one in the last year.

**Table 1.** Respondents’ demographics (n = 119) separated by swine industry experience, with respondents from the general public (public respondents; n = 66) and respondents with industry experience (industry respondents; n = 53)

Survey question	Response category	Public respondents (n)	Industry respondents (n)
Gender	Male	35	27
	Female	30	26
	Prefer not to say	1	0
Age	18–25	14	12
	26–35	23	19
	36–45	18	15
	46–55	6	4
	56–65	4	3
	66+	1	0
United States region of residency	Northeast	15	4
	Southeast	5	4
	Midwest	21	27
	West	20	16
	Southwest	5	2
Community Type	City/Urban	24	16
	Suburban	29	11
	Rural	13	26
Education Level	Less than a high school degree	0	0
	High school degree or equivalent	4	1
	Some college	14	0
	2-year degree	6	1
	4-year degree	24	26
	Master’s degree	10	11
	Professional degree	4	4
	Doctorate	4	10

### Q1. Are you aware that surgical castration may occur in the US swine industry?

**Figure 1.**
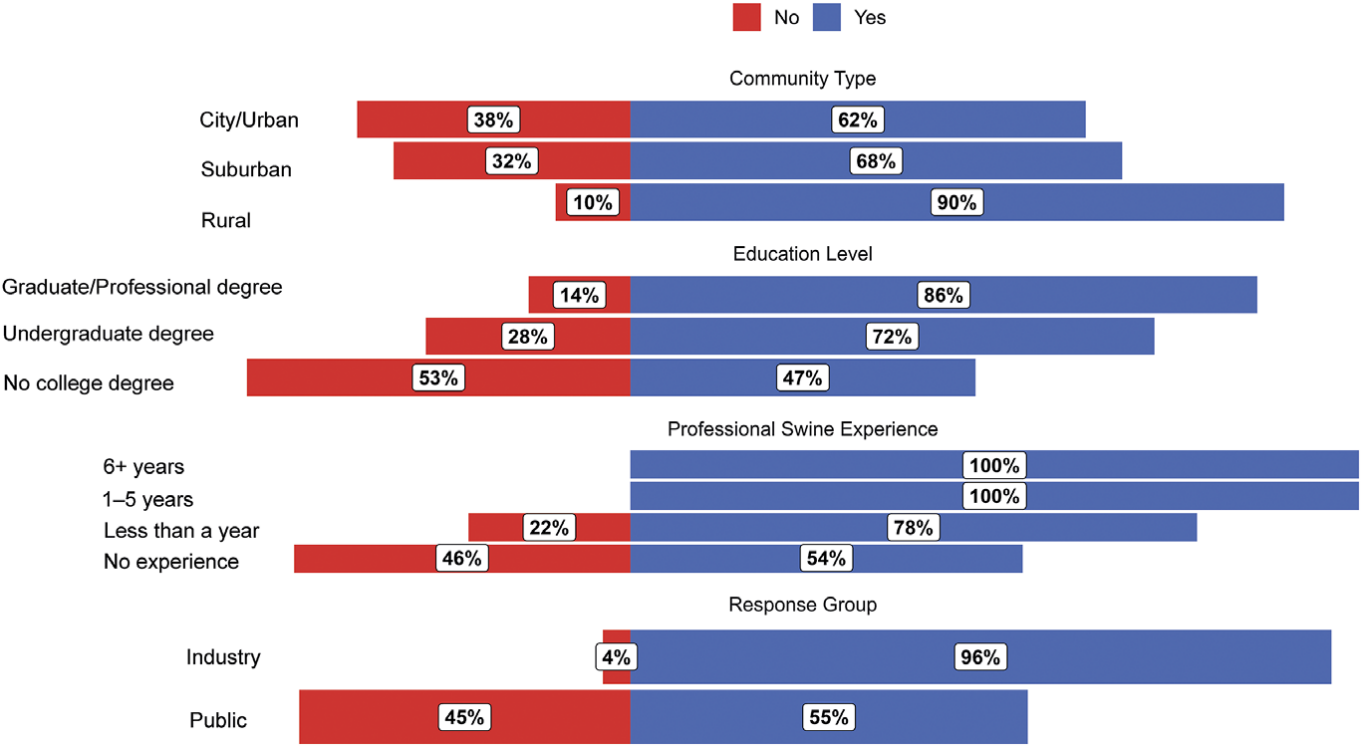
Diverging bar chart for raw responses (proportion [%] of respondents in each category) to Question 1: “Are you aware that surgical castration may occur in the US swine industry?” Respondents are categorised by ‘community type’, ‘education level’, ‘professional swine experience’, and response group (industry or public).

Respondent answers to Q1 are shown in Figure 1. Industry respondents were more aware of surgical castration in the US compared to public respondents, with industry respondents having 0.05 times the odds of selecting ‘No’ than public respondents (99% CI: 0.007–0.34; *P* < 0.001). Model predictions indicated that 3.8% (99% CI: 0.6–20.1%) of industry respondents answer ‘No’ and 96.2% (99% CI: 79.9–99.4%) answer ‘Yes’, compared to 45.5% (99% CI: 30.6–61.2%) of public respondents predicted to answer ‘No’ and 54.5% (99% CI: 38.8–69.4%) answer ‘Yes’.

In the sample of survey respondents, a higher proportion of respondents from rural communities were aware of castration compared to those from other communities. Model predictions showed 4.21 times the odds of selecting ‘No’ for suburban compared to rural community respondents (*P* = 0.022), and 3.75 times the odds for selecting ‘No’ for urban compared to rural community respondents (*P* = 0.031). Suburban and urban respondents did not differ (*P* = 0.800). The model predicted that 30.0% (99% CI: 16.2–48.7%) of urban, 32.5% (99% CI: 16.8–53.5%) of suburban, and 10.3% (99% CI: 2.9–30.8%) of rural respondents answer ‘No’, while 70.0% (99% CI: 51.3–83.8%), 67.5% (99% CI: 46.5–83.2%), and 89.7% (99% CI: 69.2–97.1%) answer ‘Yes’, respectively.

In our sample of respondents, people with higher levels of education were more aware of surgical castration. The model showed 2.85 times the odds of a ‘No’ in respondents with no college degree compared to those with an undergraduate degree (*P* = 0.055), and 6.85 times the odds compared to those with a graduate or professional degree (*P* = 0.002). Additionally, the model predicted 2.41 times the odds of a ‘No’ in respondents with an undergraduate degree compared to those with a graduate degree (*P* = 0.097). The model predicted that 52.6% (99% CI: 25.4–78.4%) of respondents with no college degree, 28.1% (99% CI: 15.4–45.5%) of respondents with an undergraduate degree, and 14.0% (99% CI: 5.0–33.5%) of respondents with a graduate or professional degree answer ‘No’, while 47.4% (99% CI: 21.6–74.6%), 71.9% (99% CI: 54.5–84.6%), and 86.0% (99% CI: 66.5–95.0%) answer ‘Yes’, respectively.

Swine industry experience impacted respondent answers to Q1 (Fisher’s Exact Test: *P* < 0.001) yet predicted probabilities and odds could not be reliably estimated.

### Q2. How often do you think castration occurs within the industry?

**Figure 2.**
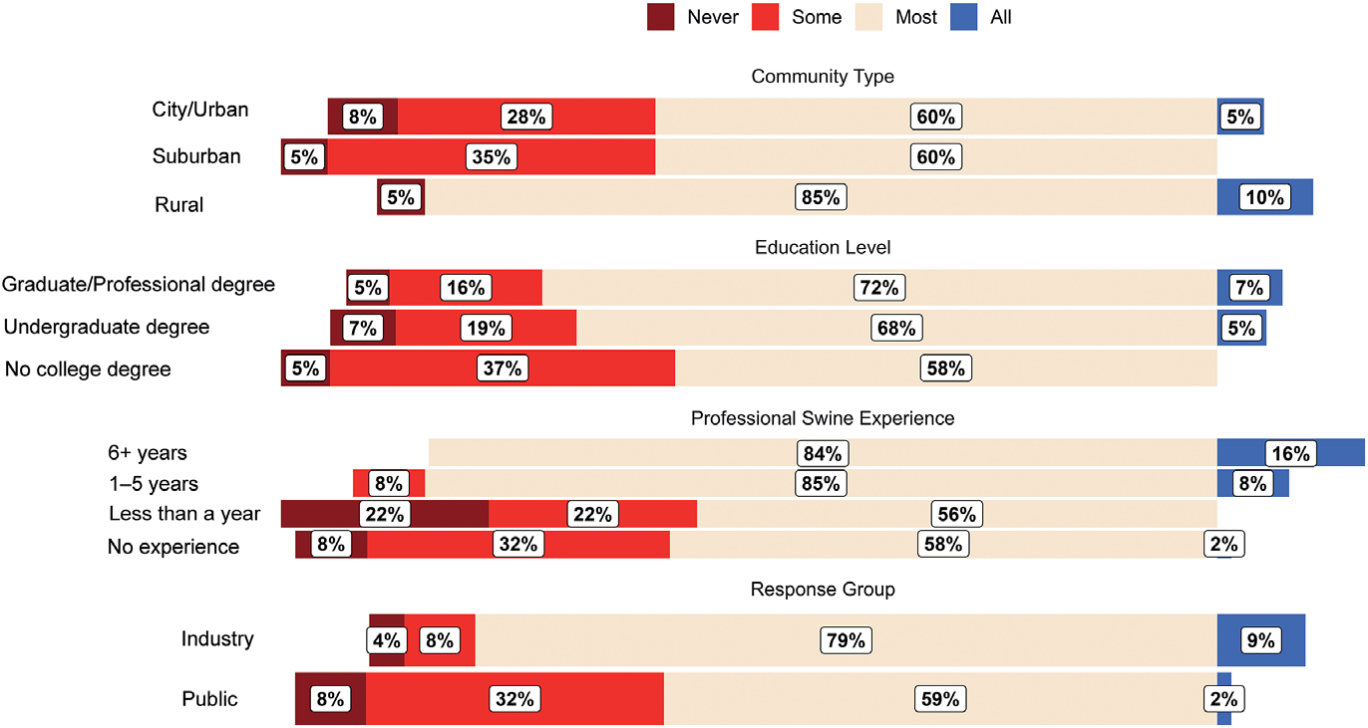
Diverging bar chart for raw responses (proportion [%] of respondents in each category) to Question 2: “How often do you think castration occurs within the industry?” Respondents are categorised by ‘community type’, ‘education level’, ‘professional swine experience’, and response group (industry or public).

Respondent answers to Q2 are shown in Figure 2. Industry respondents were more aware of the frequency that castration occurs than public respondents, with industry respondents having 0.20 times the odds of selecting a frequency less than ‘All [males are castrated]’ compared to public respondents (99% CI: 0.06–0.65; *P* < 0.001). Model predictions indicated that 9.0% of industry respondents answer ‘All’ (99% CI: 0.0–18.3%) compared to 1.9% (99% CI: 0.0–4.5%) of public respondents.

Community type impacted Q2 responses, with suburban (OR: 8.64; 99% CI: 1.8–42.4; *P* < 0.001) and urban (OR: 6.57; 99% CI: 1.3–32.2; *P* = 0.002) respondents having greater odds of selecting a frequency less than ‘All’ compared to rural respondents. Model predictions indicated that 11.7% (99% CI: 0.0–23.9%) of rural respondents answer ‘All’ compared to 1.5% (99% CI: 0.0–3.9%) for suburban and 2.0% (99% CI: 0.0–5.1%) for urban respondents. Education level did not impact responses to Q2 (*P* ≥ 0.079 for all comparisons between Education levels).

Professional swine experience impacted responses to Q2, with less industry experience increasing the odds of respondents not choosing ‘All’. Respondents with no experience had 8.46 (99% CI: 1.39–51.5) times the odds of selecting a frequency less than ‘All’ compared to respondents with 1–5 years’ experience (*P* = 0.002) and 24.0 (99% CI: 2.6–221.9) times the odds compared to respondents with more than six years’ experience (*P* < 0.001). Respondents with less than one year experience had 13.3 (99% CI: 1.2–141.0; *P* = 0.005) and 37.7 (99% CI: 2.5–561.0; *P* < 0.001) times the odds of selecting a response less than ‘All’ compared to respondents with 1–5 years’ experience or more than six years’ experience, respectively. Respondents with no or less than one year experience did not differ in their response to Q2 (*P* = 0.51), and respondents with 1–5 years and with more than six years’ experience did not differ in their responses to Q2 (*P* = 0.21). Model predictions indicated that 0.9% (99% CI: 0.0–2.6%) of inexperienced respondents, 0.6% (99% CI: 0.0–2%) of respondents with less than one year experience, 7.2% (99% CI: 0.0–18.5%) of respondents with 1–5 years’ experience, and 18% (99% CI: 0.0–39.8%) of respondents with more than six years’ experience answer ‘All’.

### Q3. Do you think any type of pain relief is routinely used during surgical castration in the US swine industry?

**Figure 3.**
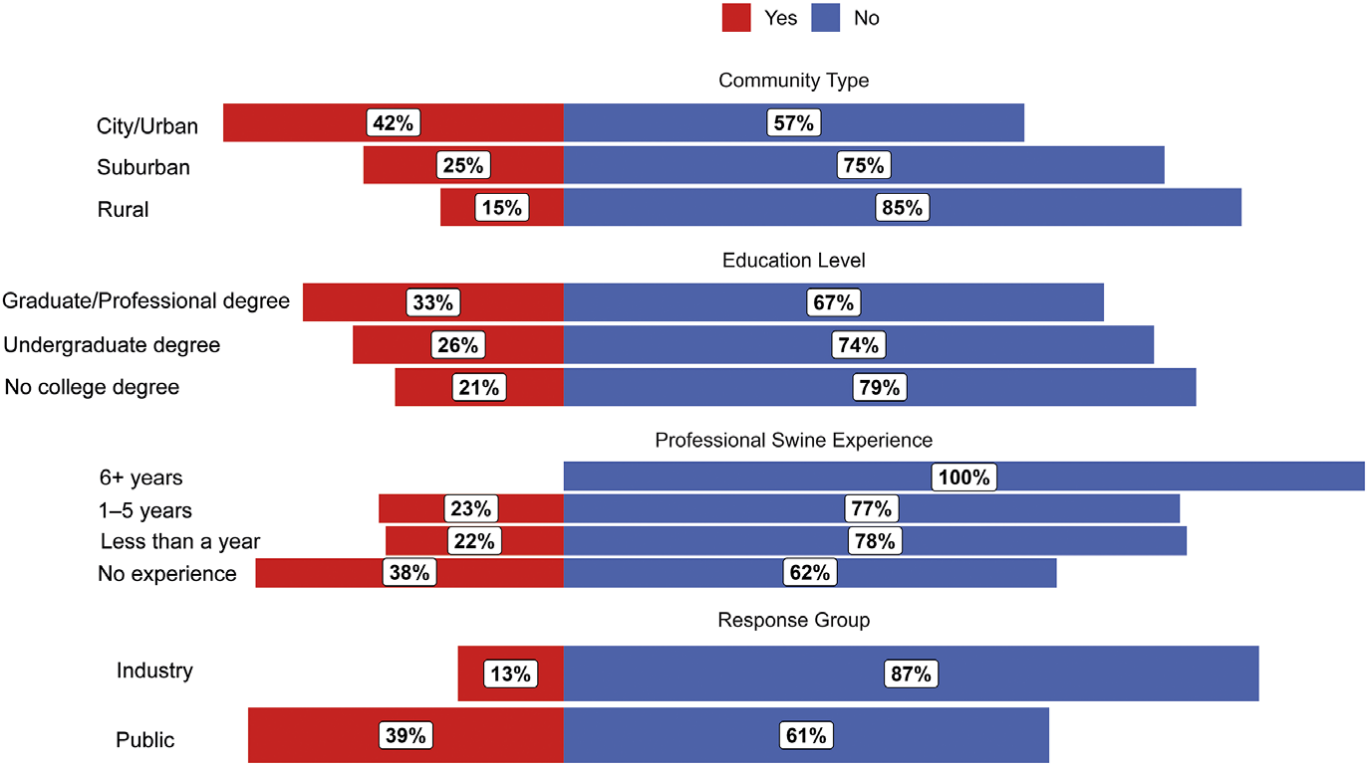
Diverging bar chart for raw responses (proportion [%] of respondents in each category) to Question 3: “Do you think any type of pain relief is routinely used during surgical castration in the US swine industry?” Respondents are categorised by ‘community type’, ‘education level’, ‘professional swine experience’, and response group (industry or public).

Respondent answers to Q3 are shown in Figure 3. Industry respondents were more aware of the lack of pain relief during castration in the US compared to public respondents, with industry respondents having 4.27 times the odds of selecting ‘No’ than public respondents (99% CI: 1.3–14.6; *P* = 0.002). Model predictions indicated that 86.8% (99% CI: 69.8–94.9%) of industry respondents answer ‘No’ and 13.2% (99% CI: 5.1–30.2%) answer ‘Yes’, compared to 60.6% (99% CI: 44.6–74.6%) of public respondents predicted to answer ‘No’ and 39.4% (99% CI: 25.4–55.4%) answer ‘Yes’.

In the sample of survey respondents, a higher proportion of respondents from rural communities were aware of the lack of pain relief than respondents from other communities. Model predictions showed 0.25 times the odds for selecting ‘No’ for urban compared to rural community respondents (*P* = 0.010). Suburban and rural respondents did not differ (*P* = 0.292) and suburban and urban respondents did not differ (*P* = 0.101). The model predicted that 57.5% (99% CI: 37.2–75.5%) of urban, 75.0% (99% CI: 53.9–88.5%) of suburban, and 84.6% (99% CI: 63.7–94.5%) of rural respondents answer ‘No’, while 42.5% (99% CI: 24.5–62.8%), 25.0% (99% CI: 11.5–46.1%), and 15.4% (99% CI: 5.5–36.3%) answer ‘Yes’, respectively.

### Q4. How often would you think pain relief is used for surgical castration in the US swine industry?

**Figure 4.**
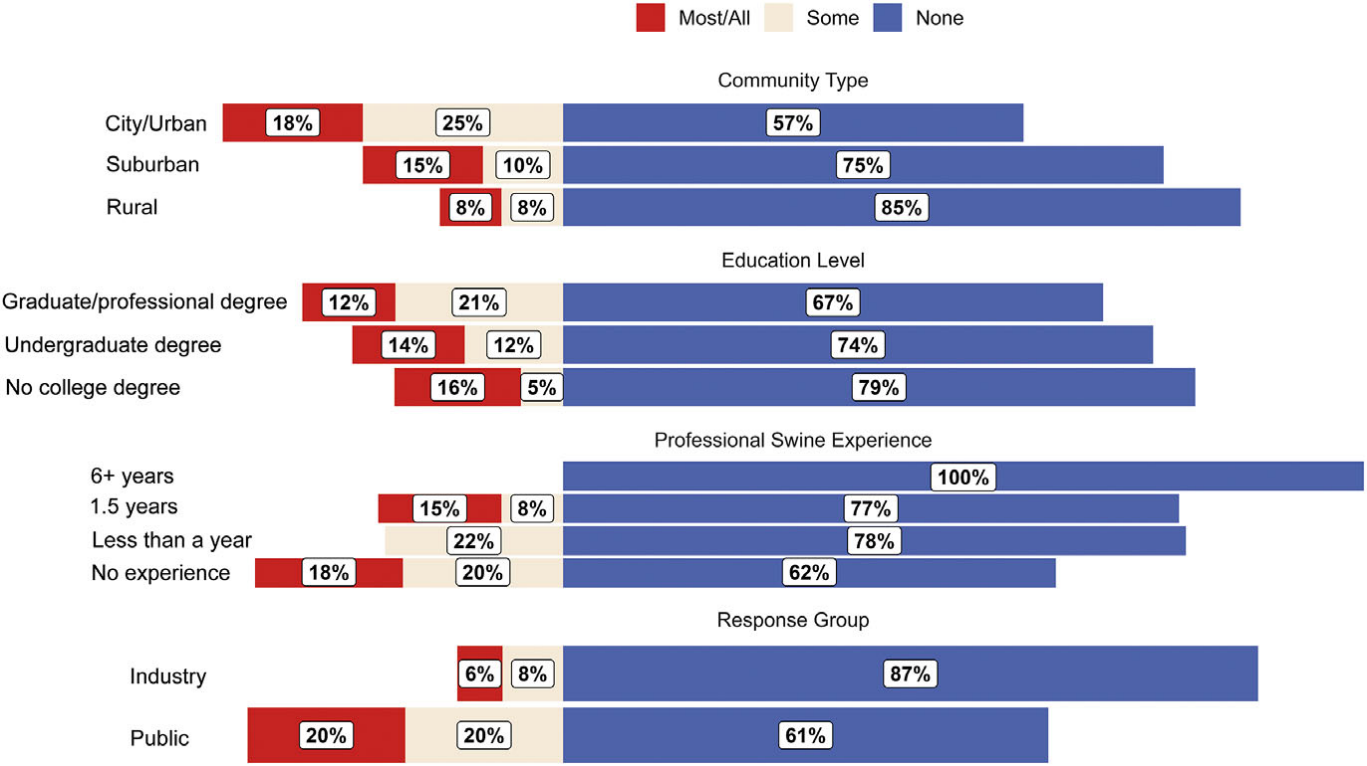
Diverging bar chart for raw responses (proportion [%] of respondents in each category) to Question 4: “How often would you think pain relief is used for surgical castration in the US swine industry?” Respondents are categorised by ‘community type’, ‘education level’, ‘professional swine experience’, and response group (industry or public).

Industry respondents were more aware of the lack of pain relief use during castration than public respondents, with industry respondents having 4.25 times the odds of selecting a frequency less than ‘Most/All [castrations used pain relief]’ compared to public respondents (99% CI: 1.3–14.4; *P* = 0.002; Figure 4). Model predictions indicated that 5.5% of industry respondents answer ‘Most/All’ (99% CI: 0.0–11.6%) compared to 19.8% (99% CI: 7.7–31.9%) of public respondents.

Community type impacted Q4 responses, with urban (OR: 0.27; 99% CI: 0.07–1.08) respondents having greater odds of selecting a frequency less than ‘Most/All’ compared to rural respondents (*P* = 0.015). Model predictions indicated that 6.7% (99% CI: 0.0–14.5%) of rural respondents answer ‘Most/All’ compared to 21.1% (99% CI: 6.0–36.1%) for urban respondents. Model predictions indicated that 12% (99% CI: 1.0–22.9%) of suburban respondents answer ‘Most/All’.

## Discussion

Pain associated with surgical castration is an animal welfare concern for piglets, and all male piglets intended for meat production are surgically castrated without analgesia in the US. As alternatives to surgical castration become more feasible to be applied in the industry, and consumer pressures could stimulate the industry to apply alternatives, greater insight is needed into what the public and industry stakeholders know about current practices. To our knowledge, this is the first study to gather information regarding the perceptions of US residents on castration practices in the US swine industry.

The survey respondents (both industry and public) were representative in terms of gender, but not in terms of age, community type, level of education, or region when compared to census data (United States Census Bureau 2019). Survey respondents were younger, more often located in suburban or rural communities, and more highly educated. In terms of region, survey respondents were mostly from the West and Midwest (30 and 40%, respectively), while census data show that those regions contain 21% of the population. This suggests that in the current study, the West and Midwest were overrepresented and respondents from the Southeast (8%) and Southwest (6%) underrepresented (United States Census Bureau 2019). The skew towards the West and the Midwest is due to swine production being concentrated in those regions (United States Department of Agriculture National Agricultural Statistics Service [USDA NASS] 2016). Survey respondents from the Northeast (16%) were sufficiently represented when compared to census data (17%; United States Census Bureau 2019). The skewed sample in this study limits the possibility of extrapolating results, and generalisating to the overall population is not appropriate, however this study does provide a first insight into respondents’ perception on US swine industry practices. This limitation could be solved by using a probability-based sampling method in future surveys.

In line with expectation, respondents with industry experience were more knowledgeable about the prevalence of surgical castration within the US swine industry. Interestingly, two industry respondents were unaware of the practice. Those individuals had indicated less than a year of professional swine experience and no experience on a sow farm, where castrations occur, explaining their lack of knowledge. Forty-five percent of public respondents were unaware of the practice, which is similar to previous findings in other countries. A minority of participants of a focus group in Norway (% not reported) were aware of routine castration of Norwegian piglets (Fredriksen *et al.* 2011). Additionally, their survey indicated that 60% of Norwegian consumers were unaware of the practice (Fredriksen *et al.* 2011). Similarly, a Belgian survey showed that 51% of respondents were unaware that male piglets are castrated (Vanhonacker *et al.* 2009). In that study, participants were recruited through non-probability snowball-sampling, and they completed the survey online. The majority (70%) of Brazilian respondents (recruited at a Brazilian airport) were unaware that surgical castration was the most common method of castration, and 76% of respondents were unaware that all male pigs slaughtered in Brazil are castrated (Yunes *et al.* 2019). The same research group surveyed the Brazilian public via an online survey (recruited through social media) and found that 77% of respondents were unaware that male pigs undergo castration or immunocastration (Hötzel *et al.* 2020). Similarly, 200 US residents surveyed about their perceptions regarding the ideal pig farm (open-ended question), only one mentioned castration as a concern (Sato *et al.* 2017). The low number of public respondents knowledgeable about surgical castration in our survey is in line with public alienation from livestock production and associated practices (Harper 2001). It was previously argued that industry stakeholders should communicate with the public, for instance by opening farm gates, by actively communicating the systems’ advantages, and by showing transparency regarding limitations (Weible *et al.* 2016). Dialogue between industry stakeholders and the general public can lead to a better understanding of the concerns and limitations of both parties (Weible *et al.* 2016).

Low levels of awareness do not have to be associated with low levels of concern. In a meta-analysis of consumer studies, the public generally shows a desire to improve farm animal welfare, regardless of the species or welfare issue under consideration (Clark *et al.* 2017). Therefore, producers and other stakeholders in the industry should be prepared to adjust their surgical castration practices to more welfare-friendly alternatives in order to maintain their social licence (Alonso *et al.* 2020).

Sixty-one percent of public respondents and 89% of industry respondents indicated that most or all male pigs are castrated, which is the answer that somewhat aligns with current US industry standards, as all commercial males intended for meat production are castrated to meet USDA Food Safety and Inspection Services regulations (Rault *et al.* 2011). Similarly, respondents with no or less than one-year experience in the swine industry were less aware of the frequency of castration taking place, compared to respondents with 1–5 years or more than six years’ experience in the industry. The survey respondents with industry experience could have considered breeding animals or niche market animals when responding to this question, explaining the ‘most’ rather than ‘all’ response from the majority of these respondents. One public respondent (2%) and five industry respondents (9%) indicated that all males are castrated, which fully aligns with industry practice. Forty percent of public respondents and 12% of industry respondents grossly underestimated the prevalence of castration in the US swine industry, when selecting ‘some[times]’ or ‘never [occurs within the industry]’ as their answers. This suggests that in both groups, knowledge is lacking as regards the widespread application of surgical castration.

Analgesic use was overestimated by both response groups, but especially the public. Thirty-nine percent of public respondents and 13% percent of the industry respondents expected some sort of pain relief to be used for piglet castration. In a conventional commercial setting, analgesics are not used for piglet castration, as none are approved for use in swine by the US Food and Drug Administration. Animal husbandry often diverges from public expectations (Weible *et al.* 2013). Furthermore, the public values other aspects of animal welfare compared to industry professionals, placing greater emphasis on ‘naturalness’, or the ability to live in a naturalistic setting, expressing natural behaviours (Skarstad *et al.* 2007; Spooner *et al.* 2014a; Thorslund *et al.* 2016; Vigors *et al.* 2021) rather than equating good health with good welfare like industry professionals (for broilers: Tuyttens *et al.* 2014, for pigs: Spooner *et al.* 2014b). These differences in values and expectations may have contributed to the public’s overestimation of analgesic use.

It was somewhat surprising that 13% of industry respondents expected analgesia to be routinely used, however it is possible that some industry respondents may have said yes because analgesia is used in their facility. Although it is not required, producers may take it upon themselves to use off-label analgesia after consultation with a veterinarian. Pain associated with surgical castration (Prunier *et al.* 2006) warrants the use of analgesia during surgical castration or the use of alternative approaches to avoid boar taint, although efficacy is not always evident (Herskin *et al.* 2016; Camerlink & Ursinus 2020). In Brazil, survey respondents showed more support for alternative approaches including analgesic use, immunocastration or raising entire males, than for surgical castration without analgesia (Hötzel *et al.* 2020). Whether the public or industry stakeholders support these alternatives in the US is not yet studied.

Respondents from rural communities were more familiar with castration practices (occurrence and prevalence) than those from other communities. In addition, they were more aware of analgesic use (occurrence and prevalence of use) than urban, but not suburban respondents. With the industrialisation of livestock production and the increasing urbanisation worldwide (Satterthwaite *et al.* 2010), the public are further distanced from agricultural practices in rural settings. This could contribute to the urban respondents’ relatively limited knowledge about farming practices.

In our survey, respondents with higher levels of education had a more accurate perception of castration prevalence in the swine industry compared to those with lower levels of education (graduate or professional degree versus no college degree or undergraduate degree, and undergraduate degree versus no degree). Yet, education level was not associated with responses to Q2–4. It should be noted that 10/14 respondents with a PhD degree were part of the industry respondent group, which likely impacted these outcomes. In line with our findings, highly educated respondents from midwestern states were more knowledgeable about agricultural practices than those with lower levels of education (Frick *et al.* 1995). In addition, respondents with higher levels of education and higher income rates demonstrated more concern for animal welfare (for a review, see Alonso *et al.* 2020). This greater level of concern could have contributed to the greater levels of knowledge about the industry or *vice versa.*

Our results highlight an opportunity for targeted education on swine industry practices especially for people with lower levels of education and people from (sub)urban communities. Although our survey specifically focused on surgical castration in the swine industry, results may indicate a wider trend for a gap in knowledge of the industry in those specific demographics. Yet, current methods do not allow for generalisation of results to the overall population or other livestock industry practices. There is no research about public or industry stakeholder knowledge of swine industry practices, or about the impact of their knowledge and perceptions on product-purchasing behaviour in the US. Yet, previous work in Europe indicates that consumers that are knowledgeable about surgical castration show aversion. For instance, willingness-to-pay for pork originating from immunocastrated pigs was 12 or 21% higher than for pork from surgically castrated pigs in Germany (Heid & Hamm 2013) and Sweden (Lagerkvist *et al.* 2006), respectively. The majority (70%) of surveyed consumers from Germany, The Netherlands and Belgium indicated a preference for immunocastration over surgical castration with analgesia (Vanhonacker & Verbeke 2011). Nearly one-third (27%) of Australian survey respondents indicated that they disapproved or strongly disapproved of piglet castration (Grahame *et al.* 2019). The aversion to surgical castration may be similar in the US and could imply that US consumers would change purchasing behaviour towards pork products originating from pigs not surgically castrated. Providing information about alternatives to surgical castration to avoid boar taint may affect public attitude (Tuyttens *et al.* 2011). Therefore, providing that information can be beneficial for public and industry acceptance of surgical castration alternatives and, in turn, improve swine welfare.

### Animal welfare implications

Surgical castration without analgesia is the most common method for preventing the development of boar taint in male piglets in the US swine industry. While there are alternatives, it is important to understand the public’s awareness of current castration practices and perceived prevalence of surgical castration and analgesia use in comparison to industry stakeholders. The present study is a first investigation into public awareness and shows that the sampled public was unaware of the process. Increased awareness can lead to informed decision-making when purchasing food, which could impact the swine industry. In addition, increased awareness could lead to rejection of the practice, as observed in other countries. Even some industry respondents were not fully aware of the castration practices in their own sector. This provides an opportunity for education or training of industry personnel related to animal welfare and related management practices. Further research should focus on a representative sample of the general public and investigate public awareness and acceptance of current castration processes and future alternatives in the US.

## Conclusion

In this study, an online survey was used to evaluate the impact of swine industry experience on survey participants’ perceptions and knowledge of US swine industry castration practices. Results indicate that public respondents were mostly unaware of castration practices and the (lack of) analgesic use. Surprisingly, a small number of industry stakeholders also showed gaps in knowledge regarding common practices. We conclude that based on this small survey, there appears to be a requirement for information and education on current castration practices and boar taint, in particular regarding respondents from (sub)urban communities and those with lower levels of education. Provision of this information can support the public in making informed decisions when purchasing products. Possible subsequent aversion to current practices, as indicated in research from Europe, could lead to consumer pressure towards alternatives to surgical castration preventing boar taint, or use of analgesia as a routine component of surgical castration. However, the current study does not allow generalisation of results to the overall population, rather it provides an initial insight into potential knowledge and awareness gaps related to swine industry practices.
